# Tissue inflammation and nitric oxide-mediated alterations in cardiovascular function are major determinants of endotoxin-induced insulin resistance

**DOI:** 10.1186/s12933-015-0223-2

**Published:** 2015-05-20

**Authors:** Lawrence M. House, Robert T. Morris, Tammy M. Barnes, Louise Lantier, Travis J. Cyphert, Owen P. McGuinness, Yolanda F. Otero

**Affiliations:** Department of Molecular Physiology and Biophysics, Vanderbilt University School of Medicine, 702 Light Hall, Nashville, TN 37232 USA; College of Medicine, University of Tennessee Health Science Center, Memphis, TN USA; Department of Biomedical Sciences, Missouri State University, Springfield, MO USA; Department of Internal Medicine, University of Michigan, Ann Arbor, MI USA

**Keywords:** Nitric oxide, Inflammation, Insulin resistance, Endotoxemia

## Abstract

**Background:**

Endotoxin (i.e. LPS) administration induces a robust inflammatory response with accompanying cardiovascular dysfunction and insulin resistance. Overabundance of nitric oxide (NO) contributes to the vascular dysfunction. However, inflammation itself also induces insulin resistance in skeletal muscle. We sought to investigate whether the cardiovascular dysfunction induced by increased NO availability without inflammatory stress can promote insulin resistance. Additionally, we examined the role of inducible nitric oxide synthase (iNOS or NOS2), the source of the increase in NO availability, in modulating LPS-induced decrease in insulin-stimulated muscle glucose uptake (MGU).

**Methods:**

The impact of NO donor infusion on insulin-stimulated whole-body and muscle glucose uptake (hyperinsulinemic-euglycemic clamps), and the cardiovascular system was assessed in chronically catheterized, conscious mice wild-type (WT) mice. The impact of LPS on insulin action and the cardiovascular system were assessed in WT and global iNOS knockout (KO) mice. Tissue blood flow and cardiac function were assessed using microspheres and echocardiography, respectively. Insulin signaling activity, and gene expression of pro-inflammatory markers were also measured.

**Results:**

NO donor infusion decreased mean arterial blood pressure, whole-body glucose requirements, and MGU in the absence of changes in skeletal muscle blood flow. LPS lowered mean arterial blood pressure and glucose requirements in WT mice, but not in iNOS KO mice. Lastly, despite an intact inflammatory response, iNOS KO mice were protected from LPS-mediated deficits in cardiac output. LPS impaired MGU in vivo, regardless of the presence of iNOS. However, ex vivo, insulin action in muscle obtained from LPS treated iNOS KO animals was protected.

**Conclusion:**

Nitric oxide excess and LPS impairs glycemic control by diminishing MGU. LPS impairs MGU by both the direct effect of inflammation on the myocyte, as well as by the indirect NO-driven cardiovascular dysfunction.

**Electronic supplementary material:**

The online version of this article (doi:10.1186/s12933-015-0223-2) contains supplementary material, which is available to authorized users.

## Introduction

Blood glucose regulation is a critical process during inflammatory states, such as blood stream infection. Improved glycemic control in the critically-ill decreases morbidity and mortality, but overly tight regulation of blood glucose poses an increased risk for hypoglycemia [1, 2]. Understanding physiologic factors driving insulin resistance in endotoxemia may direct efficacious glucose control strategies during inflammatory states.

Skeletal muscle is the tissue with the largest glucose uptake capacity in both humans and mice [3]. Impaired insulin-stimulated muscle glucose uptake (MGU) is a hallmark of insulin resistant states many of which can be induced by inflammation, obesity, diabetes, and vascular dysfunction [4–6]. Three steps are necessary for MGU: substrate delivery to muscle tissue, sarcolemmal transport, and finally myocyte metabolism (glycolysis, oxidation or storage) of glucose. Aberrations of these MGU processes can result in glucose intolerance and insulin resistance.

In the myocyte, insulin enhances MGU by driving GLUT-4 translocation to the cell membrane via insulin receptor substrate (IRS)-1, and by augmenting downstream anabolic pathways [7]. Lipopolysaccharide (LPS), a pro-inflammatory component of Gram-negative bacteria cell walls, impairs MGU by various mechanisms, including impairing IRS-1 and Akt, which are important regulators if muscle glucose transport and subsequent metabolism [5, 8–10].

LPS also has potent cardiovascular effects [11, 12]. In healthy subjects, endothelial-derived NOS is the major source of a low level of nitric oxide (NO), that is critical for normal regulation of the vascular system. During inflammation, inducible nitric oxide synthase (iNOS) activation, triggered by LPS or cytokines, generates an overabundance of NO in the circulation. High NO levels generated by LPS-mediated iNOS induce hypotension and dysregulation of tissue blood flow. Interestingly, toll-like receptor 2 activation increases systemic iNOS-derived NO without the same deleterious effects of inflammatory induction, hypotension, and limited organ perfusion seen with LPS [13]. In mice, LPS and infection compromise tissue blood flow further by impairing cardiac mechanical and electrical function (e.g. bradyarrhythmia) [14, 15]. Lastly, LPS exerts direct effects on the heart by inducing inflammation in cardiomyocytes [16, 17]. Thus, the interplay between NO abundance and systemic inflammation on cardiovascular function are incompletely understood.

Direct effects of LPS on the myocyte have been thoroughly studied. However processes upstream of cellular glucose uptake signaling machinery are emerging as important determinants of LPS-mediated insulin resistance in vivo. Cytokines [18], capillary rarefaction [19], and other processes create insulin resistance by decreasing skeletal muscle substrate delivery. Thus alteration in blood flow to skeletal muscle can indirectly limit the ability of insulin to stimulate MGU. Given the role of NO in both physiologic and pathologic states, we hypothesized that blood flow and cardiovascular factors are important determinants of MGU during endotoxemia.

To investigate the impact of nitric oxide-induced cardiovascular impairments on MGU, we utilized the hyperinsulinemic-euglycemic clamp. To simulate nitric oxide overabundance without inflammation, we infused sodium nitroprusside in wild-type (WT) mice and assessed insulin-stimulated MGU. In addition, to assess the impact of inflammation in the absence of increased NO availability we examined the impact of LPS on MGU in whole-body iNOS knock-out (KO, iNOS^−/−^) and WT control mice.

## Research design and methods

### Derivation and treatment of animals

All protocols were carried out on either wild-type (iNOS^+/+^) or global iNOS knockout (iNOS^−/−^) mice on a C57BL/6 J background (stock 002609; Jackson Laboratories, Bar Harbor, ME). Male mice were aged 2–4 months. Ambient housing temperature was 22 °C with 12 h light–dark cycles. Mice were fed *ad libitum* regular chow diet (5001 Purina Laboratory Rodent Diet) and had free access to water. Mice were handled prior to the date of experiments to minimize stress. All protocols for animal use and euthanasia were approved by the Institutional Animal Care and Use Committee at Vanderbilt University School of Medicine and were in accordance with the National Institutes of Health guidelines.

### Experimental design

**Fig. 1:**
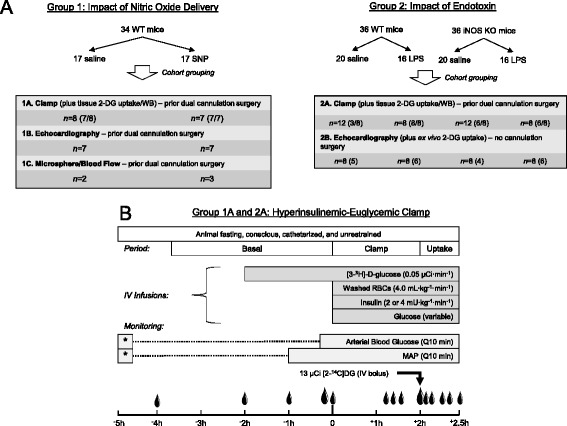
Experimental Schematic. The impact of nitric oxide delivery (*Group 1*) in wild type (*WT*) mice and LPS (*Group 2*) administration in WT and iNOS knockout mice on insulin action (hyperinsulinemic-euglycemic clamp), and cardiovascular function (echocardiography and microspheres) (**a**) was assessed. Cohorts received either a nitric oxide donor (*Group 1*), LPS (2.0 mg/kg; *Group 2*), or saline. Design of hyperinsulinemic-euglycemic clamp protocol (*Group 1* and *2*, Panel **b**) in chronically catheterized (carotid artery and jugular vein) conscious mice. Droplets indicate blood sampling times (10 μL or 25 μL of whole blood for small and large, respectively). *DG* deoxyglucose, *IV* intravenous, *KO* whole-body iNOS knockout, *LPS* lipopolysaccharide, *MAP* mean arterial blood pressure, *RBCs* red blood cells, *SNP* sodium nitroprusside

Two groups of studies were performed (Fig. 1a). The first group (Group 1) examined the effect of increased nitric oxide availability on metabolic and cardiovascular parameters. The second group (Group 2) examined the impact of LPS on these parameters in WT and iNOS knockout mice.

The first group consisted of three separate cohorts of wild type (WT) mice in which the impact of the infusion of a nitric oxide donor on insulin action (1A), cardiac function (1B) and tissue blood flow (1C) was assessed. In this group WT animals received a variable-rate infusion of sodium nitroprusside (SNP), a NO donor molecule (~37 μg · kg^−1^ · min^−1^), into the jugular vein to achieve mean arterial blood pressure (MAP) of 70 mmHg or saline. In Cohort 1A the SNP infusion was initiated 90 min before onset of hyperinsulinemic-euglycemic clamp (Fig. 1b) to assess the impact of SNP on MGU. In a subgroup, the duration of the insulin infusion was extended and the SNP was discontinued during the clamp to determine the reversibility of the effect of the SNP infusion on insulin action. In Group 1B, cardiac function was assessed using echocardiography prior to and during a SNP infusion. In Group 1C the impact of SNP on tissue blood flow was assessed using microspheres.

In Group 2, the role of iNOS on blood pressure, glucose homeostasis, and cardiac function in response to LPS was assessed. WT and iNOS KO mice were assigned to one of two treatment groups: saline (SAL) or *E. coli* endotoxin (LPS; E. coli 011:B4; Sigma-Aldrich, St. Louis, MO). Five hours after the injection of either LPS (2.0 mg/kg BW) or saline, mice were then subjected to hyperinsulinemic-euglycemic clamp (Group 2A). In a separate cohort (Group 2B), the impact of LPS on cardiac function was assessed using echocardiography in WT and iNOS KO mice. Cardiac function was assessed prior to injection of LPS and again at 3 and 5 h after LPS injection. After echocardiography at 5 h soleus muscle was excised and ex vivo insulin-stimulated MGU was determined.

### Surgical procedures

To allow us to assess the impact of SNP or LPS on blood pressure, tissue blood flow, and insulin action in conscious unstressed mice, catheters (carotid artery and jugular vein) were inserted 4–5 days prior to an experiment. While under anesthesia with isoflurane, the right jugular vein and left carotid artery were catheterized and tunneled subcutaneously to the back of the neck as previously described [20]. The catheter ends were attached via stainless steel connectors to tubing made of micro-renathane (0.033 in OD). The tubing was exteriorized, sealed with stainless steel plugs, and flushed with saline regularly to maintain patency. Animals were individually housed after surgery, and body weight (BW) was recorded the morning of each study. After insertion of catheters mice were allowed 4–5 days to regain weight within 10 % of pre-surgical body weight (BW) before undergoing in vivo experimentation.

### Hyperinsulinemic-euglycemic clamp

Hyperinsulinemic-euglycemic clamp (Fig. 1b; Group 1A and 2A) allowed measurement of whole-body insulin sensitivity and MGU as previously described [21]. In this manuscript, insulin resistance refers to decreased insulin sensitivity. This is manifest either as a decrease in whole body glucose requirements or a decrease in tissue specific glucose uptake during the clamp. Chronically catheterized conscious mice were fasted for 5 h (from *t* = −300 to 0 min) prior to insulin and glucose infusion. A bolus of 5 μCi [3-^3^H]-D-glucose was given after LPS (*t* = −120 min) followed by a 0.05 μCi/min infusion in the jugular vein catheter throughout the remainder of the study. After a 2 h equilibrium period of [3-^3^H]-D-glucose infusion, the insulin clamp was initiated with infusion of insulin, glucose, and reconstituted red blood cells (*t* = 0 min). Insulin (Novolin U-100, Novo Novonordisk Inc., Princeton, NJ, USA) was infused at a rate of 4.0 mU · kg^−1^ · BW^−1^ · min^−1^ for saline-treated mice. To yield similar clamp insulin values between groups, LPS-treated animals received insulin at a lower rate (2.0 mU · kg-1 · BW^−1^ · min^−1^) because LPS decreases insulin clearance [22]. Blood glucose was measured in blood obtained from the carotid artery every 10 min by glucometer (Accu Chek Aviva, Roche Diagnostics, Basel, CH). 50 % Dextrose (Hospira Inc., Lake Forest, IL, USA) was infused (*t* = 0 min) to maintain blood glucose at 120 mg/dL. Red blood cells from donor animals were washed with 10 % heparin saline twice then reconstituted 1:1 in 10 % heparin saline. The cells were infused continuously during the clamp (5–6 μL/min) to replace blood collected during the clamp. After the 2 h clamp period (*t* = 120 min) a 5 μCi jugular bolus of ^14^C-2-deoxyglucose ([2-^14^C] DG) was given. Blood samples for tracer analysis were collected at the beginning of the clamp and in the steady state (*t* = 80–120 min). Arterial blood samples were also collected at *t* = −10, 120, and 145 min, centrifuged and the plasma was saved for analysis of plasma insulin. At *t* = 145 min, animals were terminally anesthetized by venous injection of sodium pentobarbital (50–70 mg/kg). Soleus, gastrocnemius, superficial vastus lateralis, white adipose tissue, heart, liver, and brain were excised and freeze clamped in liquid nitrogen. Tissues and plasma were stored at −80 °C for later analysis.

To assess tracer-determined tissue glucose uptake freeze-clamped tissues obtained at the end of the clamp were homogenized in 0.5 % perchloric acid on ice. Homogenate was spun at 4000 rpm for 20 min in refrigerated centrifuge. Supernatant was neutralized to pH of 7.5 and centrifuged again at 4000 for 10 min [2-^14^C] DG and ^14^C-2-deoxyglucose-6-phosphate ([2-^14^C] DGP) contents were assessed via scintillation counting. To isolate [2-^14^C] DG from the homogenate, [2-^14^C] DGP was precipitated by addition of 0.3 N barium hydroxide and 0.3 N zinc sulfate. Precipitate was spun at 4000 for 5 min, and scintillation was quantified. Disintegrations per min (dpm) for homogenate containing only [2-^14^C] DG was subtracted from the dpm of the homogenate containing both [2-^14^C] DG and [2-^14^C] DGP to yield dpm of [2-^14^C] DGP in tissue. Tissue specific clearance (K_g_) of [2-^14^C] DG and an index of glucose uptake (R_g_; μg · min^−1^ · g^−1^) was calculated as previously described [23]:

$$ \begin{array}{c}\hfill {K}_g=\frac{\left[2{-}^{14}\mathrm{C}\right]\ {\mathrm{DGP}}_{tissue}}{AUC\ \left[2{-}^{14}C\right]D{G}_{plasma}}\hfill \\ {}\hfill {R}_g = {K}_gx\ {\left[ glucose\right]}_{plasma}\hfill \end{array} $$

Where [2-^14^C] DGP tissue is the [2-^14^C] DGP radioactivity (dpm/g) in the tissue, AUC [2-^14^C] DG plasma is the area under the plasma [2-^14^C] DG disappearance curve (dpm/ml · min), and [glucose] _plasma_ is the average blood glucose (μg/μL) during the experimental period (*t* = 122–150 min).

To assess plasma glucose kinetics during the clamp liquid scintillation counting was used to quantify the radioactivity of [3-^3^H]-glucose and [2-^14^C] DG in plasma samples. Whole-body glucose turnover rates were computed as previously described [24]. Plasma insulin was assessed by the Vanderbilt Hormone Assay and Analytical Services Core via radioimmunoassay. Plasma nonesterified free fatty acids (FFA) were sampled at *t* = −10 and 120 min and quantified using the HR Series NEFA-HR kit (Wako Pure Chemicals Industries, Ltd., Osaka, Japan).

### Mean arterial blood pressure (MAP)

The carotid artery catheter for each mouse was connected to a blood pressure analyzer (Groups 1 and 2; Digi-Med BPA, Louisville, KY) for the continuous measurement of mean arterial blood pressure (MAP). For NO donor studies (Group 1) an infusion (1 μL/min) via the jugular catheter of either saline or sodium nitroprusside (NO donor; 1.0 mg/ml; Sigma #13451) was initiated. Previous work has demonstrated that LPS reduces MAP by ~40 %. This study aimed to produce a similar deficit of ~40 % in MAP following administration of NO donor to mimic the blood pressure response to LPS (10 mg/kg). The blood pressure protocol was designed to establish this drop in MAP during the 1 h prior to the onset of the clamp (Group 1A). Adjustments in the NO donor flow rate (μL/min) were made accordingly to maintain MAP at approximately 70 mmHg. In parallel, we performed studies where saline was infused to mimic the fluid volume infused during the NO donor infusion studies.

### Body composition and echocardiography

Body composition (Group 2A) was assessed via an mq10 nuclear magnetic resonance analyzer (Bruker Optics, Billerica, MA). Heart rate, left ventricular internal dimensions (LVID, in mm), fractional shortening, ejection fraction, stroke volume, and cardiac output were determined in conscious mice immediately before and during SNP (Group 1B) infusion (1 and 3 h) or following LPS (3 and 5 h) injection (Group 2B) via echocardiography (Sonos 5500 system, Agilent, Andover, MA) by the Cardiovascular Pathophysiology and Complications Core of the Vanderbilt Mouse Metabolic Phenotyping Center [25]. Briefly, cardiac function was assessed by transthoracic echocardiography (Sonos 5500, Agilent, Andover, MA) with a 15-MHz high frequency linear transducer and frame rate of 100 frames/s. After the anterior thorax was shaved, mice were handled via the neck posteriorly, and an ultrasound gel was applied to the cardiothoracic region. An ultrasound probe was used to obtain M-mode echocardiographic images from the papillary muscles via the parasternal short-axis vies. Images were taken at a speed of 150 cm/s (maximal temporal resolution) for heart rate measurements. All other recordings were done using digitally recorded signals and images. Images were recorded at a depth setting of 20 mm. The following calculations were used to determine stroke volume and cardiac output (CO in μL · min^−1^) [26, 27]:

$$ \begin{array}{l}EDV=LVI{D}_d^3\cdot \left(\frac{7}{2.4+LVI{D}_d}\right)\hfill \\ {}ESV=LVI{D}_s^3\left(\frac{7}{2.4+LVI{D}_s}\right)\hfill \\ {}\mathrm{S}\mathrm{troke}\kern0.5em \mathrm{V}\mathrm{olume}=\mathrm{E}\mathrm{D}\mathrm{V}-\mathrm{E}\mathrm{S}\mathrm{V}\hfill \\ {}CO = \frac{Stroke\kern0.5em  Volume\cdot Heart\kern0.5em  Rate}{Body\kern0.5em  Weight\cdot 1000}\hfill \end{array} $$

where EDV and ESV are end diastolic and systolic volumes, respectively. LVID_d_ and LVID_s_ are left ventricular internal dimensions during diastole and systole, respectively. All volume measurements are expressed in microliters (μL).

### Ex vivo muscle glucose uptake (MGU)

Tracer-determined glucose uptake in excised soleus muscle was performed as in previous studies [28]. After 5 h of in vivo LPS (or saline) exposure (Group 2B), animals were anesthetized and soleus muscles (right and left) were rapidly removed. Following a 15 min basal incubation in a KRH buffer with pyruvate (12 mg/ml) and mannitol (91 mg/ml) in 95 % O_2_: 5 % CO_2_, tissues were transferred to fresh media either with or without insulin (10 mU/ml) for 30 min. Then tissues were incubated for 10 min in the same media containing non-radiolabeled 2-deoxy-D-glucose (1 mmol/L), 2-[1,2-^3^H] deoxy-D-glucose (0.25 μCi/ml), and D-[1-^14^C] mannitol (0.16 μCi/ml) either with or without insulin. Muscles were lysed, the supernatant was neutralized, and radioactivity was quantified using liquid scintillation counting.

### Immunoblotting

Skeletal muscle protein extraction was performed as in previous studies [22]. Briefly, tissues frozen at −80 °C were homogenized on ice in a 50 nM Tris buffer containing 1 mM EDTA, 1 mM EGTA, 10 % glycerol, 1 % Triton X-100, at pH 7.5, and then 1 mM DTT, 1 mM PMSF, 5 μg/ml protease inhibitor cocktail, 10 mg/ml trypsin inhibitor, 50 mM NaF, and 5 mM NaPP was added the day of extraction. Protein in tissue extract was quantified using the BioRad Protein Assay (Hercules, CA). 25 μg of protein and BioRad Precision Plus Protein Kaleidoscope Standard were size-fractionated in 4–12 % or 10 % SDS-Polyacrylamide gel. Proteins and standard molecular weight ladder were transferred to PVDF membranes. Membranes were rinsed with 1.0 % Tween in Tris-buffered saline (TBS-T) and blocked with 5 % nonfat dried milk in TBS-T. Membranes were incubated overnight at overnight at 4 °C with primary antibodies: phosphor-tyrosine, IRS-1, IRβ, phospho-Akt (Ser473), phospho-Akt (Thr308), Akt, phospho-glycogen synthase kinase (GSK-3). After the TBS-T wash, membranes were incubated with corresponding secondary anti-rabbit or anti-mouse peroxidase conjugated antibody. The proteins were detected with a chemiluminescence system (GE Healthcare, Little Chalfont, UK) exposed to X-ray film (GE Healthcare). βsystem (or in (GE Hea was used for loading control. All antibodies used were obtained from Cell Signaling Technology (Danvers, MA). Band intensity was quantified using ImageJ software.

### Real-time quantitative PCR

Snap-frozen gastrocnemius, heart, and epididymal white adipose tissue were homogenized in TRIzol (Ambion RNA, Carlsbad, CA) and extracted mRNA. cDNA was obtained from 2 μg RNA with the High Capacity cDNA Reverse Transcription kit (Applied Biosystems, Foster City, CA) and stored at −20 °C. PCR amplification through BioRad CFX system and Taqman probes for *Interleukin-6* (IL-6), *Serpine-1* (plasminogen activator inhibitor (PAI)-1), *Tumor necrosis factor* (TNF)-α, *Nitric oxide synthase 2* (iNOS), and *Chemokine (C-C motif) ligand 2* (MCP-1) allowed mRNA quantification using the ΔΔC_t_ method. Gene expression was normalized using GAPDH as a housekeeping gene. Data are R_q_ normalized to iNOS^+/+^+SAL group or R_q_ = 2^−ΔΔCt^ [ΔC_t_ = C_t_ (target)-C_t_ (GAPDH); ΔΔC_t_ = ΔC_t_ (sample)-ΔC_t_ (iNOS^+/+^+SAL)].

### Tissue blood flow (microspheres)

A separate group (Group 1C) of NO donor or saline-treated mice received a para-amino hippuric acid infusion (PAH; 24 μg/g/min) and microspheres to assess tissue blood flow. Arterial blood was collected to measure blood PAH [29]. Chronically-catheterized, conscious mice were injected with 100 μl of microspheres (Dye-Trak VII, Triton Technology, INC, San Diego, CA) followed by 50 μl of saline into the carotid artery. After 5 min mice were anesthetized, and tissues (hind limb muscle, liver, kidneys, small intestine including duodenum, jejunum and ileum) were excised. The tissues were digested overnight in 1 M KOH at 60 °C and centrifuged. The pellet containing the microspheres was washed with TritonX-100, ethanol containing 0.2 % (v/v) HCl, and finally re-suspended in ethanol. The resulting product was centrifuged, and cellosolve acetate (250 μL) was added to the microsphere:ethanol solution to elute the fluorescent dye from the microspheres; the absorbance was determined at 440 nm.

PAH clearance was used to estimate renal blood flow. The PAH clearance (ml/kg/min) was calculated as follows:

$$ PAH\  clearance=\frac{PAHi}{\left[PAH\right]}\ast \frac{1}{\left(1-Hct\right)} $$

Where PAHi is the PAH infusion rate (mg · kg^−1^ · 1H ^−1^); [PAH] is the arterial plasma PAH concentration (mg/ml) and Hct is the hematocrit ratio.

Tissue (hepatic artery, small intestine and hindlimb) blood flow was calculated as the product of the ratio of microspheres in a tissue, relative to microspheres in both kidneys and PAH clearance.

### Statistics

Paired data in the NO donor infusion experiment was analyzed with two-tailed Student’Pa*t* test. Two-way ANOVA for repeated measures data with Holm-Sidak *post hoc* analysis was used to compare groups. Whole-body insulin sensitivity was assessed via linear regression of tracer-determined whole body glucose turnover (R_d_, in mg · kg^−1^∙min^−1^) against plasma insulin levels (ng/ml). Baseline and clamp coordinates were plotted for WT receiving saline (*n* = 11) or LPS (*n* = 9) bolus, iNOS^−/−^ mice receiving saline (*n* = 8) or LPS (*n* = 8) bolus, and WT mice receiving saline (*n* = 8) or NO donor (*n* = 8) infusion. Baseline R_d_ was averaged from values assessed at 0 min and −10 min, and clamp R_d_ was averaged from four values from 80–120 min. Slope (insulin sensitivity) and ordinate-axis intercept (insulin-independent glucose disposal) for groups were compared via 95 % confidence intervals in a mixed-effect model. Data are presented as mean averaged from four values from 80–120 min. S either SigmaPlot 12.0 (Aspire Software International, Ashburn, VA) or JMP 10.0 (SAS Institute, Cary, NC). Values of *P* < 0.05 were considered statistically significant.

## Results

### NO donor lowers blood pressure and peripheral glucose uptake (Group 1A)

**Fig. 2:**
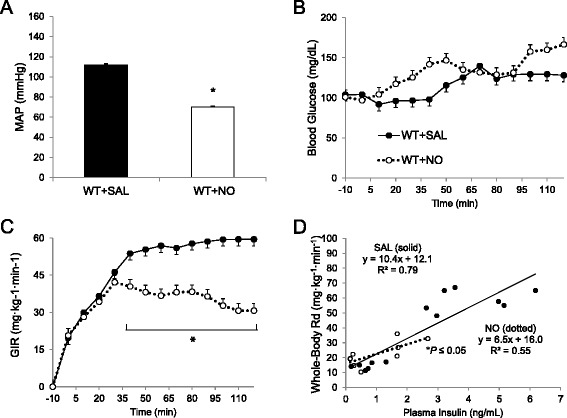
Mean arterial blood pressure (*MAP* in mmHg, Panel **a**), blood glucose (mg/dL, Panel **b**) and glucose infusion rate (*GIR* in mg · kg^−1^ · min^−1^, Panel **c**), for saline-treated (*SAL*) or sodium nitroprusside (*NO*)-treated wild-type (*WT*) mice during a hyperinsulinemic-euglycemic clamp (*Group 1A*). Mice were treated with NO 90 min prior to clamp onset (*t* = 0 min). Glucose (50 %) was infused to maintain euglycemia at 120 mg/dL during steady state (*t* = 80–120 min). Insulin sensitivity was assessed as rate of whole body glucose turnover (R_g_) per animal plasma insulin level (**d**). Data are expressed as mean ± SEM (*n* = 4–7). *p < 0.05 vs. WT + SAL. Comparison of MAP (30–120 min) and GIR (80–120 min) were assessed via *t*-test

**Fig. 3:**
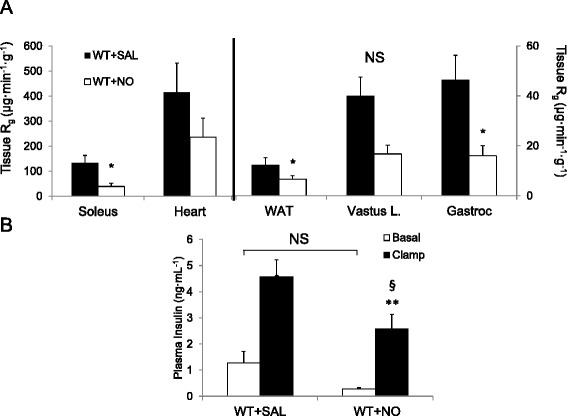
Tissue-specific tracer-determined glucose uptake (R_g_) for soleus, heart, gastrocnemius (*Gastroc*), superficial vastus lateralis (*Vastus L*), epididymal white adipose tissue (*WAT*) was assessed in wild-type (*WT*) mice 25 min after a 5 μCi bolus of ^14^C-2-deoxyglucose at the end of hyperinsulinemic-euglycemic clamp (**a**) (*Group 1A*). Plasma insulin was assessed at the onset (*t* = −10 min) and 2 h after hyperinsulinemic-euglycemic clamping (**b**). *P < 0.05 vs. WT + SAL via *t*-test. **p < 0.05 vs. WT + SAL at Clamp via *t*-test. §*P* <0.05 vs. Basal for same group via *t*-test. *NS* not significant (*p* ≥ 0.05)

Sodium nitroprusside infusion decreased MAP during the insulin clamp (Fig. 2a). Basal blood glucose and plasma insulin levels were similar between treatment groups (Table 1). During the insulin clamp, the glucose requirements (i.e., GIR) were 40 % lower for NO donor-infused mice vs. saline (Fig. 2c). However, clamp insulin levels for NO-treated mice were lower than saline-treated mice (Fig. 3b). Insulin sensitivity (slope) was lower for NO donor than saline treatment (Fig. 2d). In an extended insulin clamp, withdrawal of NO donor infusion rapidly restored GIR and arterial blood pressure levels to pre-infusion values (Additional file 1: Figure S1). The anatomical sites of NO-mediated insulin resistance were localized to soleus and gastrocnemius muscles. In addition to white adipose tissue: these tissues had significantly diminished glucose uptake during the clamp (Fig. 3a). Glucose uptake in all other measured tissues was lower for NO donor-treated animals, although not statistically significant. Thus, systemic NO abundance and the accompanying hypotension decreased peripheral glucose disposal through impaired MGU.

**Table 1:** Baseline plasma glucose and insulin concentrations

Group	Blood glucose (mg/dL)	Plasma insulin (ng/ml)
NO Donor vs. Saline		
WT + SAL	104 ± 8	1.3 ± 0.4
WT + NO	101 ± 9	0.3 ± 0.1*
LPS vs. Saline; iNOS^−/−^ vs. iNOS^+/+^ Mice		
WT + SAL	96 ± 5	0.7 ± 0.2
WT + LPS	88 ± 6	0.7 ± 0.2
iNOS KO + SAL	96 ± 5	1.0 ± 0.1
iNOS KO + LPS	83 ± 6**	0.7 ± 0.3

All blood and plasma measurements were sampled 10 min before clamp onset (i.e., *t* = −10 min). Data are presented as mean ± SEM. *p < 0.05 vs. WT + SAL; **p < 0.05 vs. iNOS KO + SAL. *iNOS* inducible nitric oxide synthase, *KO* knock-out, *LPS* lipopolysaccharide, *NO* nitric oxide donor (sodium nitroprusside), *SAL* saline, *WT* wild-type

### Cardiac function adapts during NO-mediated hypotension (Group 1B)

**Fig. 4:**
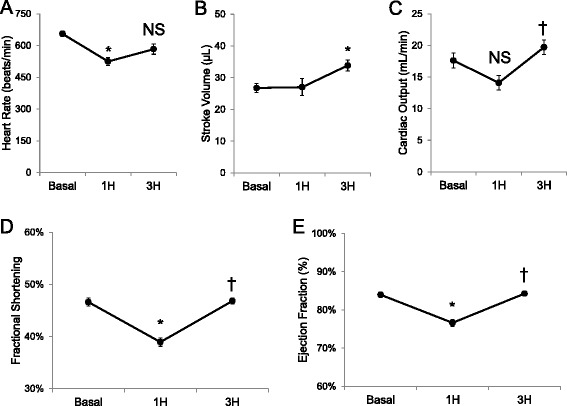
Echocardiography of wild-type (*WT*) mice 30 min before (*Basal*) and 1 h and 3 h after continuous sodium nitroprusside infusion (*Group 1B*). Data include (**a**) heart rate (in beats/min), (**b)** stroke volume (in μL), (**c**) cardiac output (in ml/min), (**d**) fractional shortening (%), and (**e**) ejection fraction (%), and. Data are expressed as mean ± SEM (*n =* 3–7). *p > 0.05 vs. Basal, *p* > 0.05 vs. 1H, *NS* not significant (*p* ≥ 0.05)

Cardiac function initially fell, but recovered during NO donor infusion (Fig. 4). Heart rate fell after 1 h vs. baseline (Fig. 4a). Both fractional shortening and ejection fraction diminished after 1 h of infusion, but both recovered at 3 h (Fig. 4d & e). Cardiac output increased significantly after 3 h of NO donor infusion (Fig. 4c). The time period of maximal cardiac impairment (i.e., 1 h of infusion in Fig. 4) corresponded to the insulin clamp onset (i.e., *t* = 0 min; see Fig. 1). Taken together, NO donor-mediated hypotension (Additional file 1: Figure S2) impaired cardiac performance, but cardiac output recovered.

### NO donor increases blood flow to intestine, but not skeletal muscle (Group 1C)

**Fig. 5:**
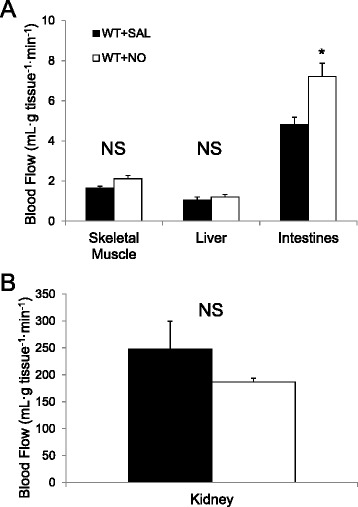
Tissue blood flow (ml · mg^−1^ · min^−1^) for muscle, liver, intestines in saline (*SAL*) or sodium nitroprusside (*NO*)-treated wild-type (*WT*) mice (**a**) (*Group 1C*). Kidney blood flow was measured as clearance (ml · kg^−1^ · min^−1^) of para-aminohippuric acid (*PAH*) clearance (**b**). Data are mean ± SEM (*n* = 3–5). *p < 0.05 vs. WT + SAL compared by *t*-test. *NS* not significant (*p* ≥ 0.05)

Since cardiac function responds dynamically to NO overabundance, we tested whether blood flow distribution to muscle was altered. Hindlimb and hepatic artery blood flow was unaltered (Fig. 5a). NO donor increased intestinal blood flow but not renal blood flow (Fig. 5a & b). Thus, NO donor infusion increased splanchnic blood flow (hepatic artery + intestine) but did not alter hindlimb blood flow.

### NO donor does not alter skeletal muscle insulin signaling (Group 1A)

**Fig. 6:**
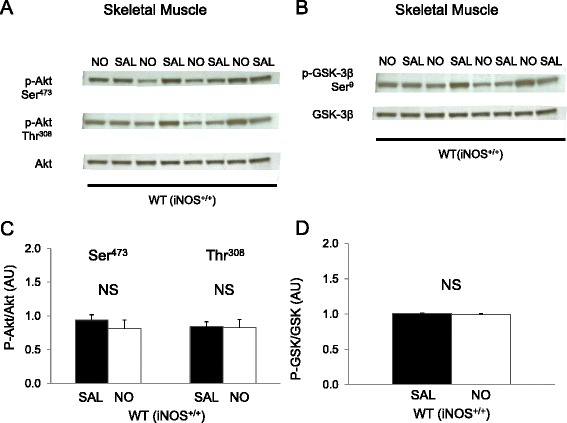
Effects of nitric oxide (*NO*) donor on skeletal muscle insulin signaling in wild-type (*WT*) mice (*Group 1A*). Western blotting was performed for plantaris muscle extract for phosphorylation (*p*) of Akt at Ser473 and Thr308 (**a**, **c**) and for glycogen synthase kinase (*GSK*) 3β phosphorylation at Ser9 residue (**b**, **d**). Chemiluminescence on 4–12 % SDS-PAGE gels was quantified for skeletal muscle. Insulin-induced protein phosphorylation was quantified as the ratio phosphorylation and total Akt or GSK chemiluminescence in skeletal muscle extract after a 2 h hyperinsulinemic-euglycemic clamp. β-actin was used as a loading control. Data are arbitrary units (*AU*) expressed as mean ± SEM (*n* = 7). *p < 0.05 by two-way ANOVA. *NS* not significant *(p* ≥ 0.05)

To determine the effects of NO donor infusion on insulin signaling in skeletal muscle, we examined downstream signaling proteins of the insulin receptor: Akt and GSK-3β from samples obtained at the end of the clamp. The infusion of NO donor during the insulin clamp did not alter Akt phosphorylation in plantaris muscle (Fig. 6a & c). Similarly, GSK-3β phosphorylation did not differ between saline and NO donor groups (Fig. 6b & d). We did not observe differences in Akt and GSK-3β phosphorylation state in soleus muscle either (data not shown).

### LPS lowers blood pressure in an iNOS-dependent manner (Group 2A)

**Fig. 7:**
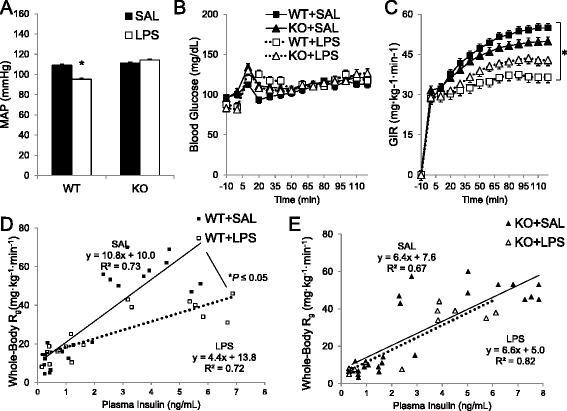
Mean arterial blood pressure (*MAP* in mmHg, Panel **a**), blood glucose (in mg/dL, Panel **b**), and glucose infusion rate (*GIR* in mg · kg^−1^ · min^−1^, Panel **c**), for saline-treated (*SAL*) or lipopolysaccharide (*LPS*)-treated iNOS^+/+^ (*WT*) or iNOS^−/−^ (*KO*) mice during a hyperinsulinemic-euglycemic clamp (*Group 2A*). Mice were treated with LPS (2 μg/g BW) 4 h prior to clamp onset (*t* = 0 min). Glucose (50 %) was infused to maintain euglycemia at 120 mg/dL during steady state (*t* = 80–120 min). Insulin sensitivity was represented as whole-body R_g_ (mg · kg^−1^ · min^−1^) vs. plasma insulin level (ng/ml) for basal and clamp time periods in iNOS^+/+^ (**d**) and iNOS^−/−^ mice (**e**). Basal glucose uptake values were determined from plasma samples at −10 min and 0 min before the onset of the clamp. Steady state (clamp) levels were determined from plasma samples at 80, 90, 100, and 120 min. MAP (**a**) was summarized as mean during end of the clamp (*t* = 60–120 min). Data are expressed as mean ± SEM (*n* = 7–8). *p < 0.05 vs. WT + SAL compared by repeated measures two-way ANOVA for MAP (30–120 min) and GIR (80–120 min)

Intravenous LPS (2 μg/g BW) decreased MAP for WT, but not iNOS KO mice (Fig. 7a). MAP did not differ between WT and iNOS KO mice prior to LPS injection (109 ± 2 vs. 111 ± 2 mmHg, respectively).

### LPS-mediated insulin resistance requires iNOS (Group 2A)

**Fig. 8:**
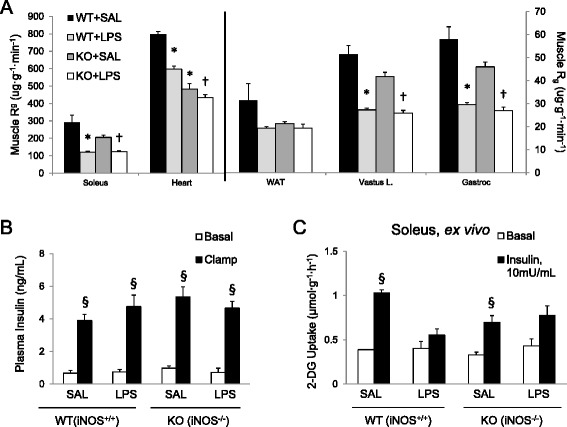
Tissue-specific insulin-stimulated glucose uptake (R_d_ in μg∙g^−1^∙min^−1^) for LPS-treated wild-type (*WT*) and iNOS knock-out (*KO*) mice (**a**) (*Group 2A*). In vivo glucose uptake was assessed 25 min after a 5 μCi bolus of ^14^C-2-deoxyglucose at the end of a hyperinsulinemic-euglycemic clamp (*n* = 3–7). Insulin levels in plasma (ng/ml) before and after the clamp (**b**). Basal and insulin-stimulated ex vivo muscle 2-DG was determined in isolated soleus (Panel **c**, *n* = 3–6). Tissues were incubated in media with or without insulin (10 mU/ml) for 30 min, then transferred to the same media containing 2-deoxy-D-glucose, 2-[1,2-^3^H] deoxy-D-glucose (2-DG, 0.25 μCi/ml), and D-[1-^14^C] mannitol (0.16 μCi/ml). Data are mean ± SEM. * *p* < 0.05 vs. WT + SAL, † *p* < 0.05 vs. KO + SAL, § *p* < 0.05 vs. Basal for same group

Basal blood glucose concentration and plasma insulin did not differ between genotypes (Table 1, Fig. 8b). LPS lowered basal blood glucose in WT mice (Table 1). Saline-treated WT and iNOS KO mice had similar glucose requirements (Fig. 7c). However, LPS decreased glucose requirements by 30 % vs. saline-treated WT (Fig. 7d & e). In iNOS KO mice, LPS treatment did not alter glucose requirements. This finding paralleled whole-body R_d_ vs. plasma insulin data: LPS mediated a ~60 % decrease in insulin sensitivity (i.e. decreased slope) for WT mice (Fig. 7d). This was not observed in iNOS KO mice (Fig. 7e). Therefore, iNOS is required for LPS-induced whole body insulin resistance. LPS increased basal free fatty acids in WT mice, but free fatty acids decreased to similar levels for all groups at the end of the insulin clamp (data not shown).

### LPS Impairs MGU Independent of iNOS in vivo (Group 2A)

Similar to NO donor-treated animals, LPS decreased MGU in soleus, gastrocnemius, and SVL for both genotypes (Fig. 8a). LPS increased IL-6 and MCP-1 in skeletal muscle in both WT and iNOS KO mice and it was higher in iNOS KO (Table 2). Additionally, LPS limited heart glucose uptake in WT mice but not in iNOS KO mice. However, R_g_ in heart muscle was lower in untreated iNOS KO than WT mice, as was the gene expression of inflammatory markers (Fig. 8a, Table 2).

**Table 2:** Impact of LPS on inflammatory gene expression in WT and iNOS KO mice

Group	Heart	Gastrocnemius
Interleukin-6 gene expression		
WT + SAL	1.5 ± 0.9	1.4 ± 0.8
WT + LPS	9.6 ± 3.2^*p* = 0.07^	61 ± 26*
iNOS KO + SAL	4.1 ± 2.3	1.4 ± 0.6
iNOS KO + LPS	19.6 ± 4.7**	9.5 ± 0.9***
Monocyte Chemoattractant Protein-1 (MCP)-1 gene expression		
WT + SAL	1.1 ± 0.4	1.1 ± 0.3
WT + LPS	2.6 ± 0.1*	4.9 ± 2.1
iNOS KO + SAL	1.8 ± 0.7	0.6 ± 0.6
iNOS KO + LPS	6.1 ± 2.1 ^*p* = 0.07^	12.1 ± 5.3**

Relative expression (R_q_) of Interleukin-6 and Monocyte Chemoattractant Protein-1 (*MCP-1*) gene measured in tissues excised at the end of a hyperinsulinemic-euglycemic clamp. Data are R_q_ values relative to the control group (WT + SAL). Data are mean ± SEM. *p < 0.05 vs. WT + SAL; **p < 0.05 vs. iNOS KO + SAL; ***p < 0.05 vs. WT + LPS. *iNOS* inducible nitric oxide synthase, *KO* knock-out, *LPS* lipopolysaccharide, *SAL* saline, *WT* wild-type

Alterations in cardiovascular function by LPS in WT but not iNOS KO mice prompted us to measure blood flow-independent MGU (Group 2B). Ex vivo insulin stimulated MGU after in vivo LPS injection allowed measurement of skeletal muscle insulin action independent of changes in substrate delivery. Similar to in vivo results*,* LPS decreased insulin-stimulated MGU in soleus for WT. While iNOS KO mice were only partially protected in vivo*,* they were completely protected ex vivo (Fig. 8c). Taken together, LPS impairs MGU both via direct effects of iNOS on the myocyte, as well as on extra-myocyte events such as substrate delivery that are determined by alterations in the vascular system.

### LPS-induced cardiac dysfunction is iNOS-dependent (Group 2B)

**Fig. 9:**
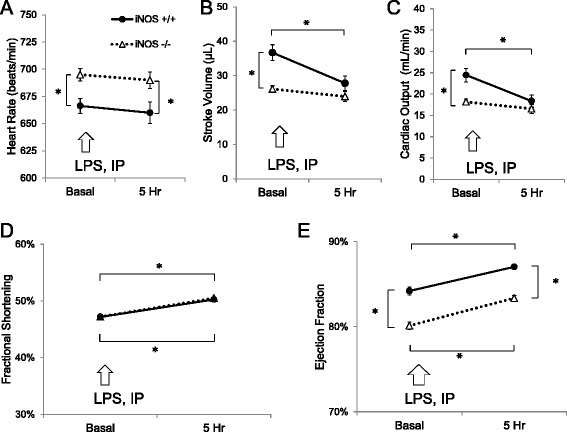
2-D Echocardiography of wild type (iNOS^+/+^) and knockout (iNOS^−/−^) mice before (*Basal*) and 5 h after intraperitoneal lipopolysaccharide (2 mg/kg LPS, IP) (*Group 2B*). Heart rate (in beats/min) (**a**), stroke volume (in μL) (**b**), cardiac output (in ml/min) (**c**), fractional shortening (%) (**d**), and ejection fraction (%) (**e**) were determined using M-mode echocardiographic measurements. Data are expressed as mean ± SEM (*n =* 7–8). * *p* < 0.05 vs. basal; or wild-type (iNOS^+/+^) vs. knock-out (*KO*; iNOS^−/−^) via two-way ANOVA

Echocardiography demonstrated genotype differences between WT and iNOS KO mice. Compared to WT mice, iNOS KO mice exhibited an elevated heart rate before and after LPS injection (Fig. 9a). Stroke volume and cardiac output were decreased by LPS injection in WT but not iNOS KO mice (Fig. 9b & c). LPS increased fractional shortening in both genotypes (Fig. 9d). While iNOS KO mice had a decreased basal ejection fraction, LPS increased ejection fraction in a parallel fashion in both genotypes (Fig. 9e).

### LPS and muscle insulin signaling in the absence of iNOS (Group 2A)

**Fig. 10:**
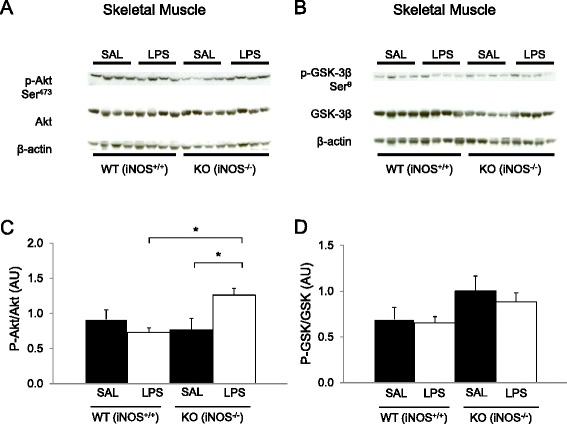
Effects of LPS on skeletal muscle insulin signaling in wild-type (*WT*) and iNOS knock-out (*KO*) mice (*Group 2A*). Western blotting was performed for gastrocnemius extract for phosphorylation (*p*) of Akt at Ser473 (**a, c**), and for glycogen synthase kinase (GSK)-3β phosphorylation at Ser9 residue (**b, d**). Chemiluminescence on 4–12 % SDS-PAGE gels was quantified for skeletal muscle. Insulin-induced protein phosphorylation was quantified as the ratio phosphorylation and total Akt and GSK-3β chemiluminescence in gastrocnemius extract after a 2 h hyperinsulinemic-euglycemic clamp. β-actin was used as a loading control. Data are arbitrary units (*AU*) expressed as mean ± SEM (*n* = 8). * *p* < 0.05 by two-way ANOVA

Similar to our previous observations [22], insulin-stimulated Akt phosphorylation was not decreased by LPS in WT mice after the 120 min insulin clamp (Fig. 10a & c). LPS treatment actually enhanced Akt phosphorylation in iNOS KO mice. Activation of skeletal muscle insulin receptor, insulin receptor substrate-a (IRS-1), and glycogen synthase kinase (GSK)-3β did not differ among groups (Fig. 10b, d and Additional file 1: Figure S3).

### LPS Induces gene expression of pro-inflammatory markers (Group 2B)

LPS increased iNOS gene expression in heart (1.0 ± 0.04 vs 5.8 ± 1.3; *p* < 0.05), gastrocnemius (1.0 ± 0.2 vs 3.7 ± 1; *p* < 0.05) and white adipose tissue (1.0 ± 0.1 vs 3.5 ± 1; *p* = 0.07) of WT mice. LPS increased the expression of IL-6 and MCP-1 expression in heart and gastrocnemius in both WT and iNOS KO mice (Table 2). In fact, in iNOS KO mice cardiac inflammation was amplified. LPS increased IL-6, but not PAI-1 and TNF-α, gene expression in white adipose tissue (Additional file 1: Table S1).

## Discussion

LPS and the accompanying inflammatory stress markedly impair insulin stimulated muscle glucose uptake as well as induce cardiovascular dysfunction [22]. We used the hyperinsulinemic-euglycemic clamp to determine the role of increased nitric oxide availability and the accompanying vascular effects, in modulating insulin action in vivo [30]. We observed that an increase in NO availability alone in the absence of an inflammatory stress markedly impaired insulin-stimulated MGU. Moreover, if we prevent the LPS induced increase in nitric oxide availability, we can partially protect against the impairment in MGU and cardiovascular dysfunction despite the accompanying pro-inflammatory environment. Thus, the LPS-induced insulin resistance is a result of a complex interaction of the direct result of inflammation on myocyte function, and the events occurring outside the myocyte that are impacted by effect of NO and inflammation on the cardiovascular system.

### Cardiovascular response to NO excess

To maintain a robust impact of insulin on MGU requires a healthy vascular system, which is disrupted by LPS and the accompanying increase in NO availability. Increased NO delivery to tissue had potent effects on the cardiovascular function. NO donor infusion caused persistent hypotension with transient changes in heart rate and cardiac output. Bradycardia following NO donor infusion in conscious mice was unexpected, but has been reported in anesthetized rats [31, 32]. It is possible that the prolonged infusion altered the baroreceptor response to hypotension [33, 34]. Bradycardia after LPS injection in mice has been observed, and contrasts with the response in adult human sepsis, which is marked by tachycardia [14, 35]. We used a lower dose of LPS and did not observe bradycardia, but it is possible that bradycardia may take longer (>16 h) to develop with the lower doses of LPS [36, 37]. The iNOS KO mice had small baseline differences (e.g. decrease cardiac output) that may be due to differential amounts of eNOS-derived NO [38], that may have altered the response to LPS. As expected, in response to LPS they were protected from the hypotension and the decrease in cardiac output [39]. The echocardiographic results were collected on conscious mice, which contrasts with other reports where anesthesia was used and the dose and route of LPS differed, all of which can alter the response [22, 30, 36, 40, 41]. However, the overall cardiovascular response to LPS is not solely reliant on iNOS, as other endocrine systems and altering signaling via cardiac ion channels can be activated [13, 37, 39, 42]. Not surprisingly, cardiovascular status during states of NO excess appears dependent upon the presence of concomitant inflammation. It is also possible that iNOS can serve a protective role [43]. However on balance, excess NO derived from iNOS has important effects on the cardiovascular response to LPS.

### Inflammation, NO availability and control of glucose uptake

Increased NO availability and LPS injection impaired insulin stimulated whole body glucose uptake. Both SNP and LPS decreased insulin action by ~50 %. Unequal insulin levels between groups can undermine the interpretation of clamp data. To account for this, we represented whole body insulin sensitivity as the slope of whole-body glucose turnover (R_g_) vs. plasma insulin. This simple, yet novel *xy* plot is useful in depicting insulin action and can facilitate interpretation of the hyperinsulinemic-euglycemic clamp data. From this relationship, both LPS (10.8 vs 4.4; Saline vs LPS) and SNP (10.4 vs 6.5; Saline vs. SNP) decreased the slope (i.e. induced insulin resistance). Moreover, iNOS KO mice were insulin resistant at baseline and LPS did not further decrease insulin action. Interestingly, baseline cardiac output was also decreased in iNOS KO mice, which paralleled the decrease in insulin action. Thus from a whole body point of view, insulin action is very sensitive to the cardiovascular responses seen in response to iNOS activation and to the increased NO that accompanies it.

The impairment in whole body glucose uptake by LPS and the NO donor was paralleled by a decrease in MGU. As we have observed previously with higher doses of LPS, MGU decreases in multiple muscle groups of varying fiber types, which have very different rates of glucose uptake in response to insulin [22]. While iNOS KO mice were partially protected from the LPS-induced decrease in insulin-stimulated MGU in vivo, they were completely protected ex vivo. The contrast between the in vivo and ex vivo data highlights the impact factors outside the myocyte that are present in vivo in determining MGU. One such signal could be a decrease in tissue blood flow seen in vivo, which would be absent in the ex vivo setting. Given the marked alteration in the vascular system by LPS and systemic NO delivery tissue flow would be expected to be altered. Microcirculatory flow and capillary recruitment are integral processes controlled by insulin, inflammation, and eNOS [6, 8, 44–47]. Manipulation of skeletal muscle capillary density and tissue flow can impair MGU [19]. Surprisingly, despite the hypotension, NO donor infusion did not decrease hind limb blood flow. Alterations in microvascular blood flow may still be present. The microspheres would not detect alterations in microcirculatory blood flow nor would it detect if the flow was “shunt or non-nutritive” flow, which could bypass the myocyte [9, 48].

### Impairment in MGU and insulin signaling

Despite marked decrease in MGU with LPS and NO donor in vivo insulin signaling was intact. This is similar to what we have previously reported, but another study did observe a decrease in muscle insulin signaling using much higher (20×) doses of LPS [22, 49, 50]. On the surface, it was surprising that the decrease in insulin stimulated MGU was not associated with a decrease in IRβ, IRS1, or Akt activation in muscle. Normal insulin signaling despite marked activation of inflammatory gene expression is also surprising. But this dissociation between signaling and MGU is manifest in iNOS KO mice, which were partially protected with no detected attenuation of inflammation or enhancement of insulin signaling. Exposure of animal to LPS and inflammation can directly impair insulin action in muscle [18, 51]. We like others observed that LPS impaired insulin-stimulated MGU ex vivo*.* Inflammation, and oxidative stress impair the ability of NO to activate phosphatidylinositol 3-kinase (PI3-K) and mitogen activated protein kinase (MAPK) within vascular smooth muscle cells of insulin resistant rats and mice [52]. One possibility as to why insulin signaling was intact is that the alteration in tissue flow may not limit insulin delivery to the myocyte to a similar extent as that of glucose. Insulin is not metabolized by the muscle so the absolute concentration seen by the myocyte would not be altered. In contrast insulin-stimulated MGU may become flow-limited when LPS or the NO donor is infused. Thus, one could see a decrease in MGU, but a relatively intact insulin signaling. It could be that with higher doses of LPS then defects of insulin signaling can be detected [49, 50]. It is also possible since the tissue samples were taken at the end of the clamp, defects in the early activation in insulin signaling could have been missed.

The fact that the NO donor induced insulin resistance was rapidly reversed (Fig. S1) after NO donor withdrawal [53] points to processes that can be acutely restored, such as microcirculatory flow. Thus, the answer to why NO donor alone impairs MGU may lie in dysregulated microcirculatory and capillary recruitment, which are known sites of regulation of muscle insulin action [54, 55]. We examined signaling in multiple muscles with differing fiber types and the response was similar. It is likely that multiple levels of MGU control are exerted by LPS for all fiber types [30]. Further endeavors investigating NO donor-induced insulin resistance should attempt to quantify capillary recruitment. Thus, alterations in exomyocellular factors (microcirculatory flow, extracellular barriers) likely play an important role in manifesting in vivo impairment in MGU.

### Differential effect of NO and LPS on insulin clearance

Another novel finding of the present study is the mechanism behind NO donor and LPS driven changes in insulin clearance. NO donor-infusion increased insulin clearance (i.e., lowered clamp insulin levels). In contrast consistent with our prior work, LPS impaired insulin clearance [22]. Because we anticipated the decrease in insulin clearance with LPS we adjusted the insulin infusion rate to better match the groups. The insulin infusion rate for LPS-treated animals was half of that for saline-treated mice (i.e., 2.0 mU/kg · min vs. 4.0 mU/kg · min). As LPS also decreased insulin clearance in the iNOS knockout mice, it would suggest that NO in the absence of a concomitant inflammatory stress has a very different impact on insulin clearance that is present with an inflammatory stress. We observed that NO donors increased splanchnic blood flow (hepatic artery + intestinal blood flow), which likely contributed to the increase in insulin clearance. In contrast, LPS likely impairs insulin clearance by a mechanism that does not require iNOS. While low dose LPS can increase hepatic blood flow [56, 57], LPS must have some direct effects on hepatic removal of insulin to impair insulin clearance. In humans, nonspecific NOS blockade increases hepatic insulin clearance [58]. Thus, the accompanying hepatic inflammation that is seen in other diseases that affect the liver may impair hepatic insulin extraction [21].

## Conclusions

Nitric oxide is a necessary signaling molecule for insulin action and glucose disposal [59]. Endotoxemia impairs in vivo and ex vivo MGU in addition to cardiac output. Thus, acute systemic inflammation impairs MGU through both tissue inflammation and cardiovascular dysfunction. This suggests that while inflammatory events occurring in the myocyte can impair insulin action*,* in vivo the accompanying defects outside the myocyte must be targeted to effectively improve MGU.

## Additional file

Additional file 1: Table S1. Gene expression of pro-inflammatory markers in white adipose tissue of wild type and iNOS knockout mice treated with LPS and after the hyperinsulinemic-euglycemic clamp. * *p* < 0.05 vs WT + SAL; † *p* < 0.05 vs KO + SAL . Figure S1. Mean arterial blood pressure (MAP in mmHg, Panel A), glucose infusion rate (GIR in mg · kg-1 · min-1, Panel B), and blood glucose (in mg/dL, Panel C) for saline-treated (SAL) or sodium nitroprusside (NO)-treated wild-type (WT; iNOS+/+) mice during experimental induction (infusion) and restoration of hypotension in an extended (180 min) hyperinsulinemic-euglycemic clamp (Group 1A). Mice were treated with IV saline or NO infusion 90 min prior to clamp onset (*t* = 0 min). Infusion was stopped at *t* = 90 min (Reversal). MAP (Panel A) was summarized as mean during infusion period (*t* = 0–60 min) and reversal period (*t* = 90–180). Data are expressed as mean ± SEM (*n* = 2). **p* ≤ 0.05 vs. WT + SAL compared by *t*-test; NS = not significant (*p* ≥ 0.05)*.* Figure S2. Continuous mean arterial blood pressure (MAP) monitoring during infusion of sodium nitroprusside (NO)-infused iNOS+/+ mice (Group 1B). Figure S3. Effects of LPS on skeletal muscle insulin signaling in wild-type (WT; INOS+/+) and iNOS knock-out (iNOS−/−) mice after a 2 h hyperinsulinemic-euglycemic clamp (Group 2A). β-tubulin was used as a loading control. Western blotting was performed for gastrocnemius and vastus lateralis extract for total and tyrosine phosphorylation of IRS1 and Insulin receptor β (IRβ).

## Abbreviations

INOS: Inducible nitric oxide synthase
NO: Nitric oxide
WT: Wild type
KO: Knock out
MGU: Muscle glucose uptake
GIR: Glucose infusion rate
LPS: Lipopolysaccharide
PAH: Para aminohippuric acid
GSK-3: Glycogen synthase kinase-3
IRS: Insulin receptor substrate
TNF-α: Tumor necrotic factor-alpha
PAI-1: Plasminogen activator inhibitor-1
TLR: Toll-like receptor
UCP-2: Uncoupling protein-2
PI3-K: Phosphatidylinositol 3-kinase
MAPK: Mitogen activated protein kinase
