# Supplementary material for: Tissue inflammation and nitric oxide-mediated alterations in cardiovascular function are major determinants of endotoxin-induced insulin resistance
Source: Cardiovasc Diabetol. 2015 May 20;14:56. doi: 10.1186/s12933-015-0223-2 (PMC4484635; doi:10.1186/s12933-015-0223-2)

Group	Interleukin- 6	TNF-a	PAI-1
WT + SAL	1.3 ± 0.6	1.2 ± 0.4	0.6±0.1
WT + LPS	8.5 ± 1.3*	1.0 ± 0.2	0.8±0.2
iNOS KO + SAL	2.4 ± 1.2	2.4 ± 0.6	0.9±0.3
iNOS KO + LPS	11.6 ± 2.7 †	1.7 ± 0.4	0.8±0.2

Table S1. Gene expression of pro-inflammatory markers in white adipose tissue of wild type and iNOS knockout mice treated with LPS and after the hyperinsulinemic-euglycemic clamp. * $p<0.05$ vs WT+SAL; † $p<0.05$ vs KO+SAL

SUPPLEMENTAL FIGURE LEGENDS

Supplement 1. Mean arterial blood pressure (MAP in mmHg, Panel A), glucose infusion rate (GIR in $mg \cdot kg^{-1} \cdot min^{-1}$, Panel B), and blood glucose (in mg/dL, Panel C) for saline-treated (SAL) or sodium nitroprusside (NO)-treated wild-type (WT; $iNOS^{+/+}$) mice during experimental induction (infusion) and restoration of hypotension in an extended (180 min) hyperinsulinemic-euglycemic clamp (Group 1A). Mice were treated with IV saline or NO infusion 90 min prior to clamp onset (t=0 min). Infusion was stopped at t=90 min (Reversal). MAP (Panel A) was summarized as mean during infusion period (t=0–60 min) and reversal period (t=90–180). Data are expressed as mean ± SEM (n=2). *$p \leq 0.05$ *vs.* WT+SAL compared by t-test; NS = not significant ($p \geq 0.05$).

Supplement 2. Continuous mean arterial blood pressure (MAP) monitoring during infusion of sodium nitroprusside (NO)-infused $iNOS^{+/+}$ mice (Group 1B).

Supplement 3. Effects of LPS on skeletal muscle insulin signaling in wild-type (WT; $INOS^{+/+}$) and iNOS knock-out ($iNOS^{-/-}$) mice after a 2 h hyperinsulinemic-euglycemic clamp (Group 2A). β-tubulin was used as a loading control. Western blotting was performed for gastrocnemius and vastus lateralis extract for total and tyrosine phosphorylation of IRS1 and Insulin receptor β (IRβ).

Supplemental Figure 1

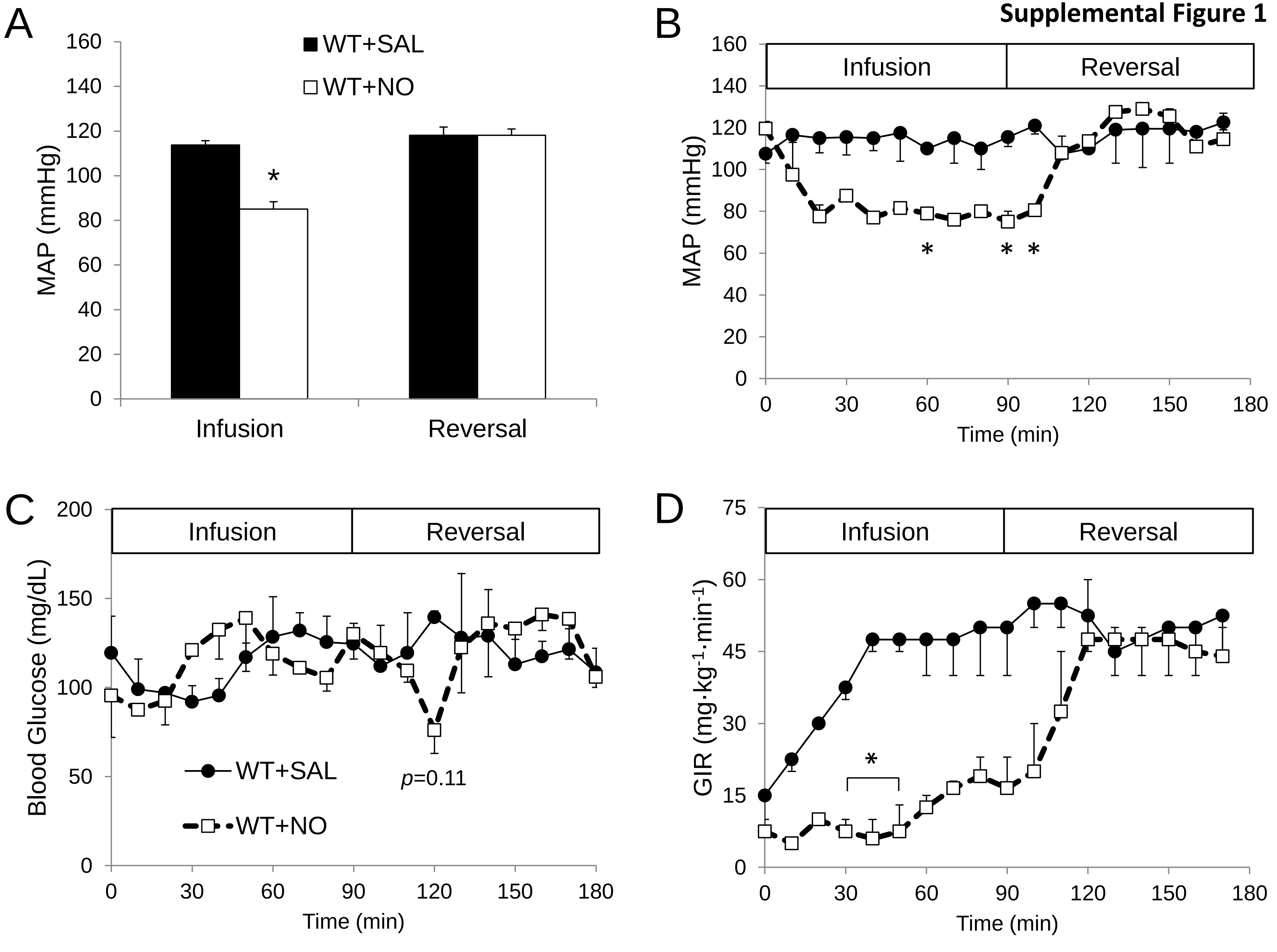

Supplemental Figure 2

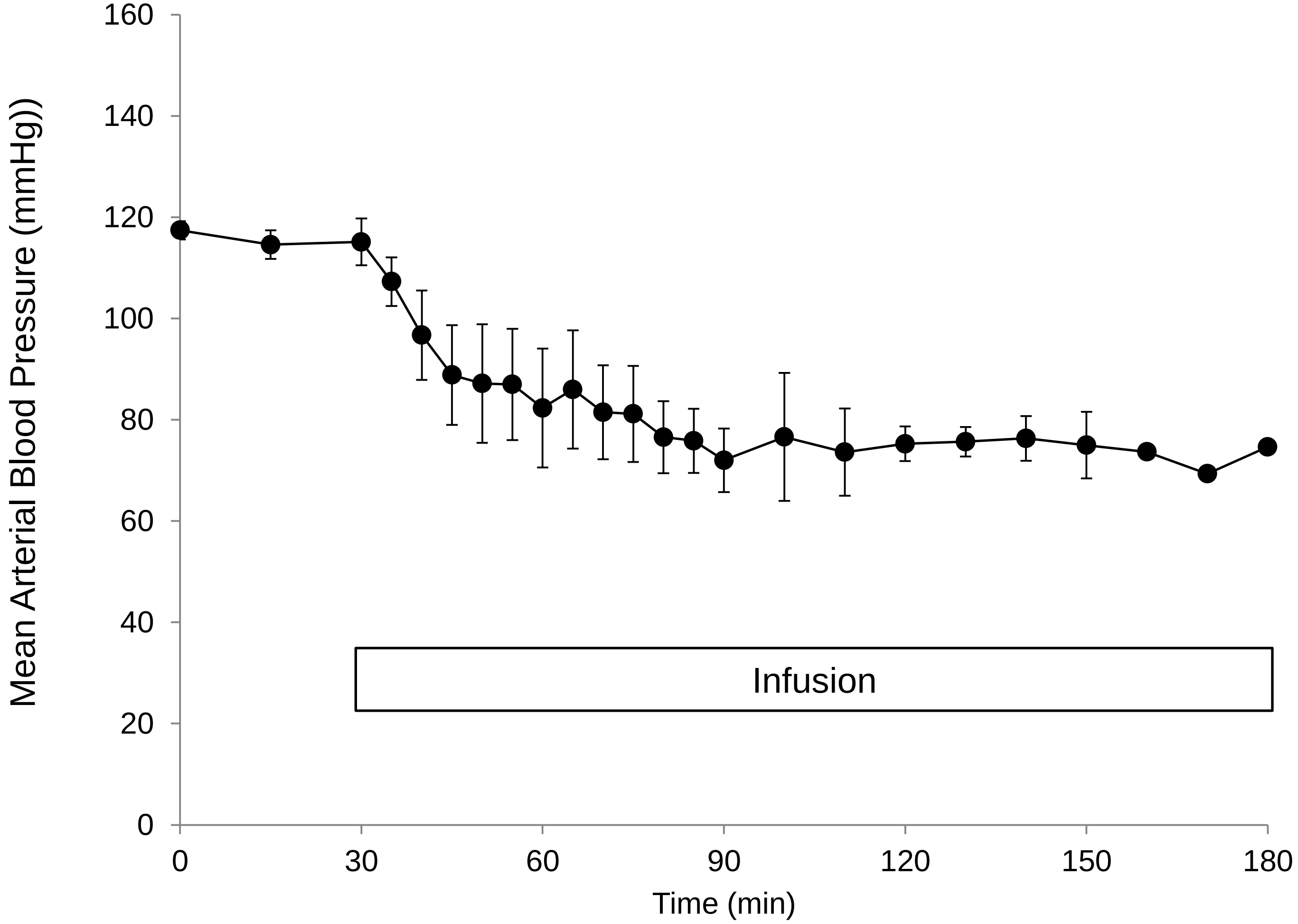

Supplemental Figure 3

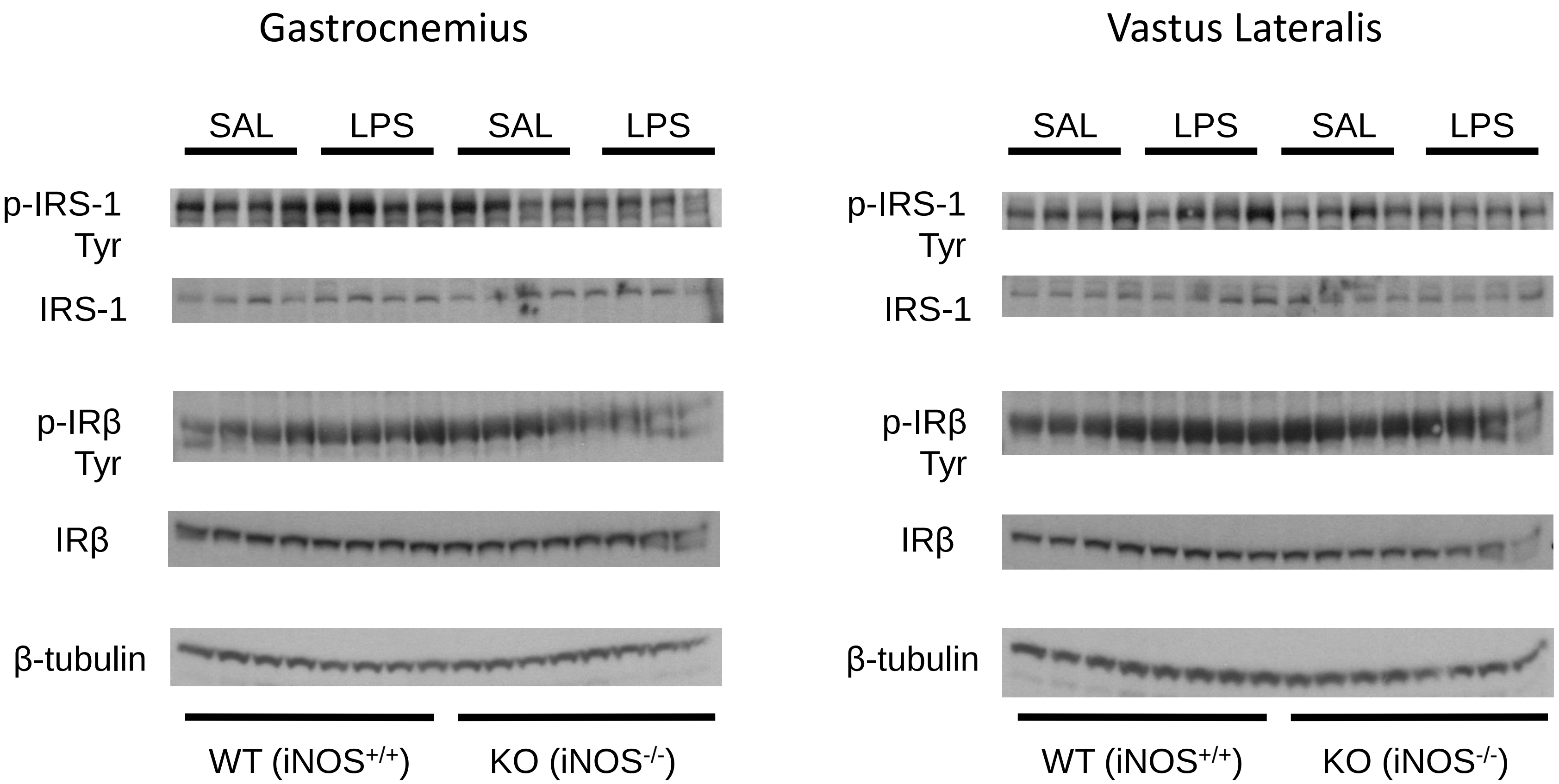

Supplement: Additional file 1: Table S1. — Gene expression of pro-inflammatory markers in white adipose tissue of wild type and iNOS knockout mice treated with LPS and after the hyperinsulinemic-euglycemic clamp. * p < 0.05 vs WT + SAL; † p < 0.05 vs KO + SAL . Figure S1. Mean arterial blood pressure (MAP in mmHg, Panel A), glucose infusion rate (GIR in mg · kg-1 · min-1, Panel B), and blood glucose (in mg/dL, Panel C) for saline-treated (SAL) or sodium nitroprusside (NO)-treated wild-type (WT; iNOS+/+) mice during experimental induction (infusion) and restoration of hypotension in an extended (180 min) hyperinsulinemic-euglycemic clamp (Group 1A). Mice were treated with IV saline or NO infusion 90 min prior to clamp onset (t = 0 min). Infusion was stopped at t = 90 min (Reversal). MAP (Panel A) was summarized as mean during infusion period (t = 0–60 min) and reversal period (t = 90–180). Data are expressed as mean ± SEM (n = 2). *p ≤ 0.05 vs. WT + SAL compared by t-test; NS = not significant (p ≥ 0.05). Figure S2. Continuous mean arterial blood pressure (MAP) monitoring during infusion of sodium nitroprusside (NO)-infused iNOS+/+ mice (Group 1B). Figure S3. Effects of LPS on skeletal muscle insulin signaling in wild-type (WT; INOS+/+) and iNOS knock-out (iNOS−/−) mice after a 2 h hyperinsulinemic-euglycemic clamp (Group 2A). β-tubulin was used as a loading control. Western blotting was performed for gastrocnemius and vastus lateralis extract for total and tyrosine phosphorylation of IRS1 and Insulin receptor β (IRβ). [file 12933_2015_223_MOESM1_ESM.pdf]
